# Primary Small-Cell Carcinoma of the Urinary Bladder: A Case Report and Literature Review

**DOI:** 10.7759/cureus.56318

**Published:** 2024-03-17

**Authors:** Carlos A Garcia-Becerra, Maria I Arias-Gallardo, Jesus E Juarez-Garcia, Veronica Soltero-Molinar, Carlos M Garcia-Gutierrez

**Affiliations:** 1 Urology Department, Urovallarta Medical Center, Puerto Vallarta, MEX; 2 Histopathology, Universidad Autonoma de Guadalajara, Guadalajara, MEX; 3 Research, Universidad Autonoma de Guadalajara, Guadalajara, MEX; 4 Anatomy, Universidad Autonoma de Guadalajara, Guadalajara, MEX

**Keywords:** bladder tumor, metastases, small cell carcinoma, urinary bladder, neuroendocrine carcinoma

## Abstract

Small-cell carcinoma of the bladder (SCCB) is an uncommon and aggressive malignancy of the urinary tract. Its clinical presentation often mimics that of other bladder neoplasms, posing a diagnostic challenge. This case report presents a rare instance of SCCB in a 65-year-old female, shedding light on the diagnostic journey and emphasizing the need for heightened and prompt clinical suspicion due to its aggressive nature. The patient presented to the urological department with hematuria, dysuria, and hypogastric pain. Initial investigations revealed a bladder mass, prompting biopsies with inconclusive results. A comprehensive histopathological examination, including immunohistochemistry, confirmed a SCCB. A computed tomography (CT) scan was used to evaluate local and distal extention. Following the initial evaluation, a referral to an oncological service was needed. Diagnoses encompassed SCCB, with interventions that comprise chemotherapy without radical cystectomy. Despite the rarity of SCCB, timely and accurate diagnosis facilitated a tailored multidisciplinary approach, leading to prompt clinical oncology management. This case demonstrates the importance of meticulous diagnostic evaluation in rare malignancies, guiding individualized therapeutic strategies for optimal patient outcomes.

## Introduction

Bladder cancer (BCa) is the tenth most common malignancy overall, with an estimated mortality rate of more than 199,000 per year worldwide; however, most of them are classified as urothelial. Non-urothelial cancers are described as a rare entity, accounting for five percent of all BCa [1,2]. Some non-urothelial carcinomas are further classified as primary neuroendocrine malignancies, which account for less than one percent of all BCa [1,2]. Small-cell carcinoma of the bladder (SCCB) is an infrequent but aggressive primary high-grade neuroendocrine malignancy of the bladder that accounts for 0.5-0.7 percent of the total BCa prevalence [2]. There is a higher prevalence of SCCB in males, with a male-to-female ratio of 5:1 [2].

SCCB resembles lung small-cell cancer in the sense that it is histologically similar, and it also sometimes develops paraneoplastic syndromes (Cushing syndrome, hypercalcemia, hypophosphatemia, etc.) [2-5].

In the present case report, the aim is to shed light on the complexities of a prompt diagnosis and the challenges associated with SCCB. The main purpose of this report is to contribute valuable insights into the diagnostic strategies employed in this rare neoplasm.

To contextualize this case report within the existing medical knowledge landscape, relevant literature was reviewed from the databases “PubMed” and “Scholar Google” using the search terms "Small-Cell Carcinoma of the Bladder," "bladder neoplasms," and "Primary Neuroendocrine Neoplasia of the Bladder."

This case is being presented with the merit of the diagnosis of a very uncommon neoplasm in a female patient as an aggressive and advanced form of SCCB that required prompt diagnosis and multidisciplinary treatment.

## Case presentation

A 65-year-old Latina woman presented to the urological department with a one-month history of gross hematuria, hypogastric pain, and dysuria. Symptoms started one month before presentation with recurrent episodes of hematuria and dysuria and episodic pain referred to the hypogastric region that sometimes radiates to the umbilical region and left renal fossa. The patient denied any remarkable past medical or surgical history; moreover, no family history of cancer is referred to by either the patient or her relatives. On physical examination, vital signs were blood pressure of 122/80 mmHg, respiratory rate of 18 per minute, body temperature of 36°C, and heart rate of 80 beats per minute. In addition to the hypogastric and umbilical areas being painful, no other signs or symptoms were observed. At the time of the presentation, hematic biometry, clinical chemistry, and urine analysis showed >100 red blood cells per high power field. The remaining laboratory tests (hematic biometry and clinical chemistry) were normal. 

**Figure 1:**
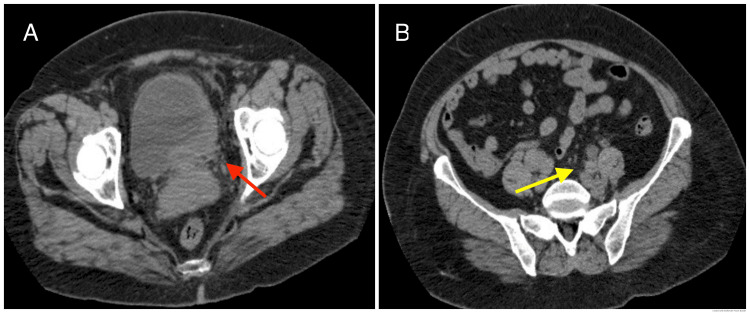
A computed tomography scan was performed for disease stratification. A: thickening of the left parasagittal aspect of the bladder (red arrow); B: common iliac suspicious adenopathies (yellow arrow).

A cystoscopy was performed after the initial assessment, where an image suggestive of non-exophytic polypoid neoplasia was found near the left ureteric orifice with a flat deformation of the typical urothelial characteristics, ulcerated, and with active hemorrhage; a biopsy was obtained and sent to histopathology for further analysis. A postoperative computed tomography (CT) scan was obtained to stratify the extension due to the high probability of deep dissemination in a flat non-exophytic malignancy. The CT scan showed thickening of the left parasagittal aspect of the bladder with secondary uretero-pyelocaliceal dilation, infiltration of the proximal vagina, suspicious bibasal lung nodules, and retroperitoneal and iliac suspicious lymphadenopathies (Figures 1A, 1B).

**Figure 2:**
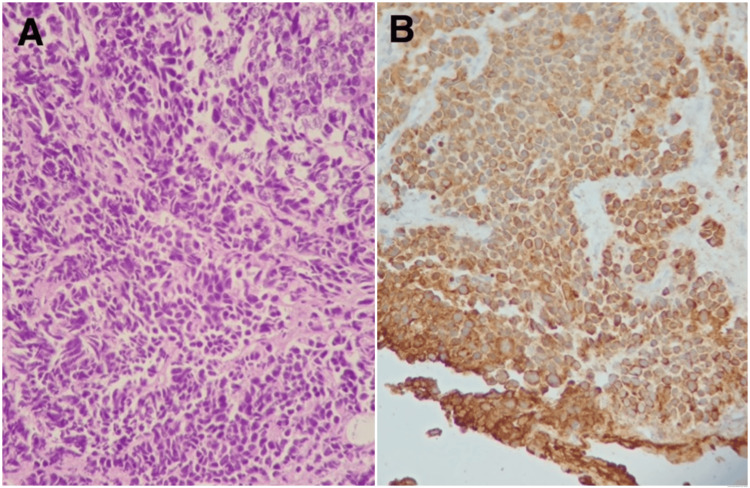
Histological paraffin block sections were performed, with histological preparation of tissue submitted as from transurethral bladder resection with hematoxylin, eosin, and immunohistochemical staining. A: hematoxylin and eosin staining (x200). B: Immunohistochemistry method for CKAE1/AE3 receptors; Chromogranin A receptors; Uroplakina III receptors; GATA 3 receptors and KI-67 protein.

After inconclusive initial histopathological analysis, further immunohistochemistry analysis was required; the sample was processed, searching for the receptors CK AE1/AE3, Chromogranin A, Uroplakin III, GATA 3, and protein KI-67. The result showed positive receptors CK AE1/AE3 (100%), Chromogranin A (100%), and KI-67 (90%), making certain the diagnosis of neuroendocrine small-cell carcinoma of the bladder (Figures 2A, 2B).

Final diagnostic: Combined with the patient’s medical history, cytoscopy, immunohistochemistry, and CT scan, the final diagnosis was made, concluding an invasive SCCB with a TNM stage of T4aNxM0.

Treatment, outcome, and follow-up: A multidisciplinary approach was needed; the patient was referred to an external clinical oncology service, where she continued with tailored chemotherapy. A nephrostomy was needed in a matter of months due to severe vesical trigone deformation by the tumor, with subsequent obstructive nephropathy and hydronephrosis.

At eight months postoperative, the patient was still alive, with the nephrostomy’s catheter being periodically changed and being followed up by an external oncology service.

## Discussion

First described by Koss in 1975 [6], the SCCB is described by the literature as a very uncommon BCa type, with an overall prevalence of 0.5-0.7% [1,2,7]. Despite its prevalence, it is reported to be an aggressive malignancy because of its high metastatic potential even when the bladder lesion appears as a “small lesion” [7-9]. Usually, the image found on the mucosa of the bladder is described as a necrotic ulcerated lesion of around 4-10 cm and has been difficult to differentiate from a transitional cell carcinoma (TCC) [7,9]. The most commonly described location in the bladder is the lateral wall (54%), followed by the posterior wall, trigone, dome, and anterior wall (20%, 10%, 8%, and 8%, respectively) [7,9].

From the 286 reported cases of SCCB, the prevalence in male patients is estimated to be 84% [9]. Gross hematuria has been the most prominent symptom in around 90% of these patients; other symptoms, such as dysuria, pelvic pain, or even paraneoplastic syndromes, are less frequent [7,9]. It is estimated in some series that 94% of the cases had muscle invasion when the diagnosis was made [9], whereas metastatic diseases range, depending on the bibliography, from 67% [9] to 28-50% [7] during the disease course. The most common sites of metastatic disease are the lymph nodes, liver, bone, lung, and brain [7,9].

The present case report details the diagnostic challenge that the SCCB represents when it could be confused at the initial steps of the diagnosis with a TCC, being that it is an uncommon disease that the physician might not consider a first diagnosis and given that its nature requires a very prompt identification to avoid metastatic disease as much as possible.

The limitations of this case report primarily lie in the aggressive and advanced stage of the disease when the diagnosis was made because of the already metastatic presentation of this patient. Furthermore, this case report demonstrated the substantial need to promptly integrate the observed clinical manifestations, biopsy, immunohistochemistry analyses, radiological staging, and tailored multidisciplinary treatment to increase the probability of good results in the challenging odyssey against SCCB.

## Conclusions

Conclusively, this case report contributes valuable insights into the clinical landscape of small-cell carcinoma of the bladder. The findings underscore the need for prompt clinical suspicion, tailored therapeutic approaches, and continued research to improve the prognosis of patients facing this formidable malignancy that tends to present as an early metastatic disease. As urologists navigate the complex terrain of rare malignancies, such detailed case reports serve as vital building blocks for advancing our understanding and refining our clinical approaches.
